# Cardiovascular Complications and Subclinical Myocardial Dysfunction in Patients Undergoing Hematopoietic Stem Cell Transplantation

**DOI:** 10.3390/cancers18121871

**Published:** 2026-06-08

**Authors:** Sabina Caciolli, Andrea Grasso Granchietti, Francesco Vanni, Meghi Murati, Martina Vito, Matteo Vannini, Leandro Cosco, Giacomo Coltro, Andrea Pasquini, Chiara Nozzoli, Maurizio Pieroni

**Affiliations:** 1 Department of Cardiothoracic and Vascular Sciences, Azienda Ospedaliera Universitaria Careggi, 50134 Florence, Italy; andrea.grasso@unifi.it (A.G.G.); vannif@aou-careggi.toscana.it (F.V.); meghi.murati@unifi.it (M.M.); martina.vito@unifi.it (M.V.); vanninima@aou-careggi.toscana.it (M.V.); leandro.cosco@unifi.it (L.C.); maurizio.pieroni@unifi.it (M.P.); 2 Transplant Unit, Department of Hematology, Azienda Ospedaliera Universitaria Careggi, 50134 Florence, Italy; giacomo.coltro@unifi.it (G.C.); andrea.pasquini@unifi.it (A.P.); chiara.nozzoli@unifi.it (C.N.)

**Keywords:** hematopoietic stem cell transplantation, cardio-oncology, cardiovascular complications, global longitudinal strain, echocardiography, cardiotoxicity

## Abstract

**Background**: Cardiovascular complications are increasingly recognized in patients undergoing hematopoietic stem cell transplantation (HSCT). Early detection of subclinical myocardial dysfunction may improve risk stratification, and global longitudinal strain (GLS) is emerging as a sensitive marker of early cardiac impairment. **Methods**: We conducted a single-center observational cohort study including 518 adult patients undergoing autologous (*n* = 64) or allogeneic (*n* = 454) HSCT between 2004 and 2025. Baseline cardiovascular risk factors, transplant characteristics, and echocardiographic parameters—including GLS in a subset—were recorded. Abnormal GLS was defined as less negative than −20%. The primary outcome was the occurrence of cardiovascular events (composite of cardiovascular death, myocardial infarction, stroke, atrial fibrillation/flutter, pericardial effusion, pulmonary embolism, and left ventricular systolic dysfunction). Multivariable logistic regression was used to identify independent predictors. **Results**: Median age was 53 years; 58% were male. Cardiovascular events were predominantly atrial fibrillation, pericardial effusion, and reduced left ventricular function, whereas ischemic events were rare. Over 90% of events occurred within 100 days post-transplant. Multivariable analysis identified older age (OR 1.28 per 10-year increment; 95% CI 1.10–1.48; *p* = 0.002), chronic kidney disease (OR 2.44; 95% CI 1.18–5.02; *p* = 0.01), pre-transplant atrial fibrillation (OR 2.12; 95% CI 1.04–4.31; *p* = 0.03), and abnormal baseline GLS (OR 1.89; 95% CI 1.02–3.52; *p* = 0.04) as independent predictors. Importantly, the prognostic value of GLS remained significant after excluding clinically insignificant pericardial effusions from the composite endpoint. GLS deterioration during follow-up occurred more frequently in patients receiving reduced-intensity conditioning compared with myeloablative conditioning (25% vs. 12.7%; *p* = 0.006). **Conclusions**: Subclinical myocardial dysfunction detected by GLS identifies HSCT recipients at increased cardiovascular risk. These findings support the incorporation of strain imaging into routine pre- and post-transplant cardiovascular evaluation to enable earlier detection and guide targeted interventions.

## Simple Summary

Hematopoietic stem cell transplantation (HSCT) is a potentially curative treatment for many blood cancers and other serious hematologic diseases, but it may also expose patients to cardiovascular complications. These complications are increasingly relevant as transplant recipients are older and often have more comorbidities than in the past. In this study, we analyzed a large single-center cohort of adult patients undergoing HSCT and evaluated both clinically apparent cardiovascular events and subtle myocardial dysfunction detected by echocardiographic strain imaging. We found that most cardiovascular complications were arrhythmias, pericardial disease, or ventricular dysfunction, whereas myocardial infarctions and strokes were uncommon. In addition, subclinical myocardial dysfunction detected by global longitudinal strain occurred more frequently than overt cardiovascular events, particularly in patients receiving reduced-intensity conditioning regimens. These findings support the need for systematic cardiovascular monitoring in HSCT recipients and reinforce the role of cardio-oncology in the long-term care of patients with hematologic malignancies.

## 1. Introduction

Hematopoietic stem cell transplantation (HSCT) is a potentially curative treatment for a broad spectrum of hematologic malignancies and selected non-malignant bone marrow disorders. Improvements in transplant techniques, conditioning regimens, and supportive care have progressively expanded eligibility to patients older and with a higher comorbidity burden, but cardiovascular toxicity remains an important source of short- and long-term morbidity.

Cardiovascular toxicity has therefore emerged as an increasingly relevant component of the broader field of cardio-oncology, which focuses on the prevention, early detection, and management of cardiovascular complications associated with cancer therapies. In patients undergoing HSCT, cardiovascular risk may be amplified by multiple factors, including prior exposure to cardiotoxic chemotherapy, conditioning regimens, systemic inflammatory responses, endothelial injury, and metabolic disturbances occurring during the transplant process. As survival after HSCT continues to improve, the identification of both overt cardiovascular events and early markers of subclinical myocardial injury has become an important clinical priority in the long-term management of transplant recipients [1].

Reported rates of cardiovascular complications vary substantially across studies, likely because of differences in patient populations, transplant platforms, conditioning strategies, and outcome definitions [2]. Contemporary real-world data are therefore needed to better characterize the current incidence and determinants of cardiovascular complications in the modern transplant era.

The aim of the present study was therefore to evaluate the incidence, timing, and determinants of cardiovascular complications in a contemporary single-center cohort of adult patients undergoing HSCT between 2004 and 2025. In addition, we sought to assess the occurrence of subclinical myocardial dysfunction using global longitudinal strain (GLS), an echocardiographic marker increasingly used for the early detection of cancer therapy-related cardiac dysfunction.

## 2. Materials and Methods

### 2.1. Study Design and Population

We conducted a single-center observational cohort study including 518 adult patients (≥18 years) who underwent autologous or allogeneic HSCT at the Transplant Unit of Azienda Ospedaliero-Universitaria Careggi between January 2004 and June 2025. All data were obtained from routine standard-of-care clinical practice, and all patients had provided written informed consent for hematologic treatment and the use of their clinical data. The need for ethical committee approval was waived according to Italian law no. 11960, released on 13 July 2004, regulating observational studies. Baseline clinical evaluation was performed prior to transplantation and included demographic characteristics (age, sex), risk factors (smoking history, hypertension, diabetes mellitus, hypercholesterolemia, body mass index (BMI), chronic kidney disease, coronary artery disease, and prior atrial fibrillation), hematologic diagnosis and transplant type.

Transplant-related variables included transplant type (autologous vs. allogeneic), conditioning regimen intensity (myeloablative conditioning—MAC—or reduced-intensity conditioning—RIC), and the occurrence of graft-versus-host disease (GVHD).

Transplant-related comorbidity burden was assessed using the Hematopoietic Cell Transplantation–Comorbidity Index (HCT-CI) [3].

All patients underwent cardiovascular evaluation including transthoracic echocardiography performed according to contemporary recommendations [4]. Left ventricular ejection fraction (LVEF) was measured using the biplane Simpson method; reduced LVEF was defined as <55%. In a subset of more recently transplanted patients, myocardial deformation was assessed using two-dimensional speckle-tracking echocardiography. GLS values less negative than −20% were considered abnormal.

### 2.2. Outcomes

The primary outcome was the occurrence of CV events, defined as a composite of cardiovascular death, nonfatal myocardial infarction, stroke, left ventricular systolic dysfunction, atrial fibrillation or flutter, pericardial effusion, and pulmonary embolism. Acute kidney injury was recorded as a secondary outcome. Events were categorized according to timing as early events (≤100 days after HSCT) and late events (>100 days). Because the composite endpoint included clinically distinct cardiovascular complications, individual patients could contribute to more than one event category.

### 2.3. Statistical Analysis

Categorical variables are presented as frequencies and percentages, and continuous variables as mean ± standard deviation or median with interquartile range, as appropriate. Group comparisons were performed using the χ^2^ test or Fisher’s exact test for categorical variables and the Student’s t test for continuous variables, according to data distribution.

Multivariable logistic regression analysis was performed to identify independent predictors of cardiovascular events. Results are reported as odds ratios (OR) with 95% confidence intervals (CI). Because more than 90% of cardiovascular events occurred within the first 100 days after transplantation and exact timing for all individual events was not consistently available because of the retrospective design, logistic regression was selected as the primary analytical approach to identify predictors of event occurrence.

Sensitivity analyses were additionally performed:

1. Excluding clinically insignificant pericardial effusions from the composite endpoint;
2. Stratifying the cohort according to transplant era (2004–2014 vs. 2015–2025);
3. Comparing patients with and without available GLS data to assess potential selection bias.

A two-sided *p* value < 0.05 was considered statistically significant. Statistical analyses were performed using SPSS Statistics (IBM, USA, version 31.0.1.0).

## 3. Results

### 3.1. Study Population

Among the 518 adult patients who underwent HSCT during the study period, 454 (87.6%) received allogeneic transplantation and 64 (12.4%) autologous transplantation. Median age at transplantation was 53 years, and 58.3% of patients were male.

Smoking history was present in 44.0% of patients, hypertension in 27.6%, hypercholesterolemia in 13.3%, and diabetes mellitus in 6.0%. A BMI ≥ 25 kg/m^2^ was observed in 29.9% of patients. Chronic kidney disease and pre-existing atrial fibrillation were less frequent, occurring in 4.1% and 3.9% of patients, respectively. Baseline left ventricular systolic dysfunction was uncommon. Among the subset with available strain analysis, abnormal baseline GLS was present in 14.6%, Table 1. Because GLS assessment was progressively implemented during the later study period, GLS measurements were available in 338 patients (65.2%). To assess potential selection bias, baseline characteristics and cardiovascular event rates were compared between patients with and without available GLS data (Table 2).

No major differences in overall cardiovascular event rates were observed between patients with versus without GLS assessment (30.5% vs. 31.3%, *p* = 0.588), supporting the overall consistency of the study population despite incomplete GLS availability (Table 2).

To account for temporal changes in transplant practice and echocardiographic assessment, patients were stratified according to the transplant period (2004–2014 vs. 2015–2025).

The overall incidence of cardiovascular complications was similar between the periods (33.3% vs. 30.8%, *p* = 0.653), whereas GLS assessment became significantly more frequent in the most recent period (37.2% vs. 68.3%, *p* < 0.001) (Table 3).

These findings suggest that the overall burden of cardiovascular complications remained relatively stable despite changes in transplant strategies and imaging availability over time.

### 3.2. Underlying Hematologic Diseases and Conditioning

Acute myeloid leukemia was the most common indication for HSCT overall (44.0%), followed by acute lymphoblastic leukemia (15.6%), lymphoma (11.2%), myelodysplastic syndromes (8.5%), and myeloproliferative neoplasms (8.1%). Autologous transplantation was performed predominantly for multiple myeloma and lymphoma, whereas allogeneic transplantation was used mainly for myeloid malignancies (Table 4).

**Figure 1:**
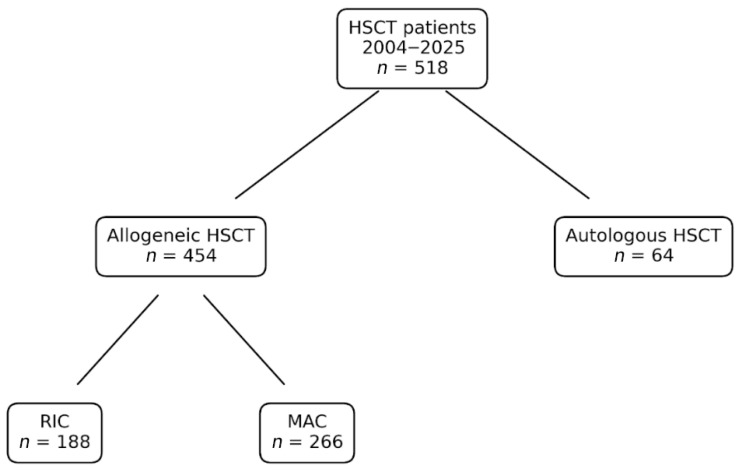
Study population flow chart.

Among allogeneic recipients, 188 patients (41.4%) received RIC and 266 (58.6%) underwent MAC. Patients treated with RIC were older than those receiving MAC and more frequently had hypertension and chronic kidney disease, whereas smoking history was more common in the MAC group (Figure 1, Table 5).

### 3.3. Incidence and Pattern of Cardiovascular Events

Overall, cardiovascular complications were characterized predominantly by atrial fibrillation, pericardial effusion, and reduced left ventricular ejection fraction, whereas myocardial infarction and stroke were uncommon. Most cardiovascular events occurred early after transplantation, with more than 90% arising within the first 100 days.

When stratified by transplant type, overt cardiovascular event rates were broadly similar between autologous and allogeneic recipients. Atrial fibrillation occurred in 17.2% of autologous recipients and 12.3% of allogeneic recipients. Pericardial effusion occurred in 10.9% and 15.9%, respectively. Reduced left ventricular ejection fraction was observed in 21.9% of autologous recipients and 13.6% of allogeneic recipients (*p* = 0.07). Myocardial infarction and stroke were rare in both groups. Acute kidney injury was the only complication significantly more frequent among allogeneic recipients than among autologous recipients (13.4% vs. 3.1%, *p* = 0.03).

**Figure 2:**
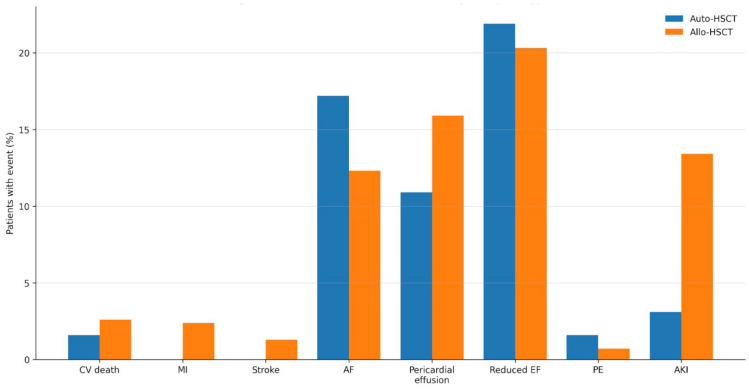
Cardiovascular and renal events by transplant type.

**Figure 3:**
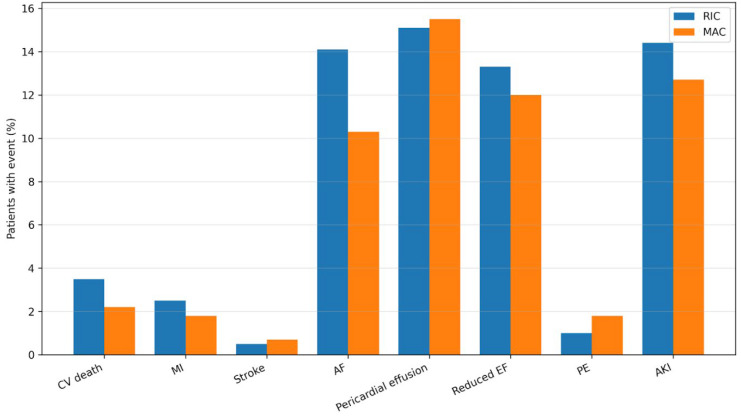
Cardiovascular and renal events by conditioning regimen.

Among allogeneic recipients, the overall incidence of overt cardiovascular events was similar between patients receiving RIC and those receiving MAC. Pericardial effusion occurred in 15.1% of RIC recipients and 15.5% of MAC recipients, atrial fibrillation in 14.1% and 10.3%, and reduced ejection fraction in 17.0% and 12.8%, respectively (Table 6, Figure 2 and Figure 3). Event-specific incidence rates are detailed in Table 3 and Table 6, with atrial fibrillation/flutter and pericardial effusion representing the most frequent cardiovascular complications.

### 3.4. Subclinical Myocardial Dysfunction

Although baseline GLS abnormalities were similar between conditioning groups, deterioration in myocardial deformation during follow-up was more frequent among patients receiving RIC. New abnormal GLS developed in 25.0% of RIC recipients compared with 12.7% of those treated with MAC (*p* = 0.006). GLS deterioration during follow-up was more frequent among patients receiving RIC despite similar rates of overt cardiovascular events (Table 7A).

### 3.5. Graft-Versus-Host Disease

The occurrence of GVHD was significantly associated with the development of cardiovascular events (OR 1.68; 95% CI 1.15–2.46; *p* = 0.008).

Among allogeneic recipients, overweight status and acute kidney injury were more common among patients who developed GVHD. Pulmonary embolism occurred more frequently in patients with GVHD (5.9% vs. 0.6%), whereas pericardial effusion was less common (7.8% vs. 18.2%) (Table 7B). No significant differences were observed in other cardiovascular outcomes, including myocardial infarction, stroke, atrial fibrillation, left ventricular systolic dysfunction, or GLS impairment.

### 3.6. Multivariable Predictors of Cardiovascular Events

In multivariable logistic regression analysis, older age was independently associated with increased cardiovascular risk after hematopoietic stem cell transplantation (OR 1.28 per 10-year increment; 95% CI 1.10–1.48; *p* = 0.002). Chronic kidney disease (CKD) (OR 2.44; 95% CI 1.18–5.02; *p* = 0.01) and pre-transplant atrial fibrillation (AF) (OR 2.12; 95% CI 1.04–4.31; *p* = 0.03) were also significant predictors.

**Figure 4:**
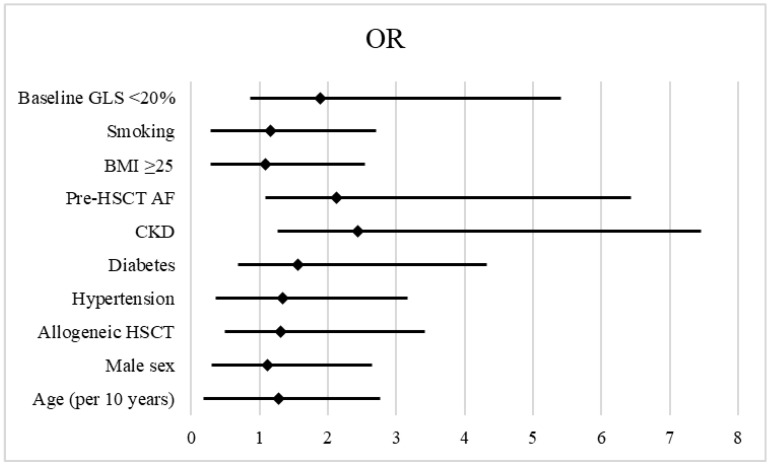
Forest plot showing multivariable predictors of cardiovascular events after HSCT.

Subclinical myocardial dysfunction, defined as baseline GLS less negative than −20%, conferred a nearly twofold higher risk of cardiovascular events (OR 1.89; 95% CI 1.02–3.52; *p* = 0.04). In contrast, male sex, allogeneic HSCT, hypertension, diabetes, BMI ≥ 25, and smoking were not independently associated, although hypertension showed a nonsignificant trend toward increased risk (OR 1.34; 95% CI 0.98–1.82; *p* = 0.07) (Table 8, Figure 4). Because the primary composite endpoint included clinically heterogeneous events, an additional sensitivity analysis excluding clinically insignificant pericardial effusions was performed.

After exclusion of asymptomatic pericardial effusions, the overall incidence of cardiovascular complications decreased from 31.5% to 27.6%. Importantly, abnormal baseline GLS remained independently associated with cardiovascular events (OR 2.34; 95% CI 1.03–5.33; *p* = 0.043), supporting the robustness of the main findings.

For subclinical myocardial dysfunction, reduced-intensity conditioning (RIC) increased the risk of GLS deterioration compared with myeloablative conditioning (OR 2.10; 95% CI 1.24–3.54; *p* = 0.006), and older age remained an independent predictor (OR 1.21; 95% CI 1.05–1.41; *p* = 0.01).

Regarding graft-versus-host disease (GVHD), overweight status conferred a significantly higher risk (OR 3.40; 95% CI 1.80–6.45; *p* < 0.001).

## 4. Discussion

In this observational cohort study including a large population of patients undergoing HSCT, we evaluated the incidence and characteristics of cardiovascular complications, with a particular focus on subclinical myocardial dysfunction assessed by GLS. Several important findings emerged.

First, cardiovascular complications after HSCT were relatively frequent but were predominantly represented by non-ischemic manifestations, including atrial fibrillation, pericardial effusion, and left ventricular systolic dysfunction, whereas major ischemic events such as myocardial infarction and stroke were comparatively uncommon. These findings are consistent with the increasingly recognized concept that cardiovascular toxicity after HSCT is largely driven by inflammatory, endothelial, and myocardial injury mechanisms rather than by accelerated atherosclerotic disease alone [2,5,6,7,8].

Second, we observed that abnormalities in myocardial deformation assessed by GLS were associated with a significantly increased risk of cardiovascular complications. Importantly, abnormal baseline GLS remained independently associated with cardiovascular events even after excluding clinically insignificant pericardial effusions from the composite endpoint, supporting the robustness of the findings and suggesting that subclinical myocardial dysfunction may identify a population at increased cardiovascular vulnerability before transplantation.

GLS has emerged as one of the most sensitive echocardiographic markers of early myocardial dysfunction in cardio-oncology [9,10,11,12]. In patients receiving potentially cardiotoxic therapies, strain abnormalities frequently precede reductions in left ventricular ejection fraction and may identify patients at higher risk for overt cardiotoxicity [13,14]. Our findings extend this concept to the HSCT population and suggest that myocardial strain imaging may help identify patients with pre-existing myocardial vulnerability who are more likely to develop cardiovascular complications during the transplant course.

Importantly, our study adopted a broader definition of cardiovascular complications compared with several previous HSCT studies. In addition to major cardiovascular outcomes, we also included events with lower immediate clinical severity, such as pericardial effusions not requiring invasive treatment. Although these events may not carry the same prognostic weight as myocardial infarction or cardiovascular death, they likely reflect early cardiovascular injury occurring during the transplant process and may therefore still be clinically relevant within the cardio-oncology setting [15,16,17].

To address the potential heterogeneity of the composite endpoint, we performed an additional sensitivity analysis excluding clinically insignificant pericardial effusions. Notably, the association between abnormal baseline GLS and cardiovascular complications remained significant after this adjustment, further supporting the consistency of the observed relationship between strain abnormalities and cardiovascular risk.

Another relevant finding was the higher incidence of GLS deterioration observed among patients receiving reduced-intensity conditioning (RIC) compared with myeloablative conditioning (MAC). However, these results should be interpreted cautiously. Patients receiving RIC were generally older and presented a higher burden of baseline cardiovascular comorbidities, potentially introducing residual confounding despite multivariable adjustment. Accordingly, the observed association between RIC and GLS worsening should be considered hypothesis-generating rather than causal [18,19].

Because GLS assessment became progressively implemented during the later years of the study period [20,21,22], strain data were available only in a subset of patients. This represents a potential source of selection bias. Nevertheless, we observed no major differences in cardiovascular event rates between patients with versus without available GLS measurements, partially mitigating concerns regarding substantial selection bias and supporting the overall consistency of the cohort.

The long duration of the study period represents both a strength and a limitation. On one hand, it allowed inclusion of a large real-world HSCT population with prolonged follow-up. On the other hand, transplant strategies, supportive care, cardiovascular surveillance, and echocardiographic techniques evolved considerably between 2004 and 2025 [23]. To address this issue, we performed an era-based sensitivity analysis, which demonstrated relatively stable cardiovascular event rates across different transplant eras despite increased availability of GLS assessment in more recent years.

Our study has potential clinical implications. Current cardiovascular surveillance strategies in HSCT recipients remain incompletely standardized, particularly regarding identification of patients at highest risk for early cardiovascular complications [24,25,26,27,28,29,30]. The present findings suggest that incorporation of GLS into routine pre-transplant echocardiographic assessment may improve cardiovascular risk stratification and facilitate earlier identification of patients with subclinical myocardial vulnerability.

In conclusion, cardiovascular complications after HSCT are predominantly characterized by non-ischemic manifestations, including arrhythmias and myocardial dysfunction. Subclinical myocardial dysfunction detected by impaired GLS identifies patients at increased cardiovascular risk and may represent an important tool for cardiovascular risk stratification in HSCT recipients.

## 5. Limitations

This study has several limitations. It was conducted at a single center and is therefore subject to the limitations of observational retrospective analysis. The study period was long, spanning important changes in transplant practice, supportive care, and echocardiographic assessment.

GLS was available only in a subset of patients, reflecting the gradual implementation of strain imaging during the study period, potentially introducing selection bias. However, sensitivity analyses comparing patients with and without GLS data showed no major differences in cardiovascular event rates.

The composite endpoint included events with heterogeneous clinical relevance. Nevertheless, sensitivity analyses excluding clinically insignificant pericardial effusions yielded consistent findings.

Given the relatively limited number of autologous HSCT recipients, comparisons between autologous and allogeneic transplantation should be considered exploratory and hypothesis-generating. Larger prospective studies are needed to better define potential differences in cardiovascular risk profiles between transplant settings.

Detailed cumulative anthracycline exposure, calcineurin inhibitor levels, melphalan exposure burden, and post-transplant cyclophosphamide prophylaxis data were not systematically available because of the retrospective nature of the study.

Finally, exact timing for all individual cardiovascular events was not consistently available, limiting the feasibility of comprehensive time-to-event analyses.

## 6. Conclusions

In this single-center cohort of HSCT recipients, cardiovascular complications were frequent and predominantly characterized by arrhythmias, pericardial disease, and subclinical ventricular dysfunction, whereas ischemic events remained uncommon.

Our findings support the use of comprehensive cardiovascular surveillance strategies that go beyond conventional LVEF assessment. In line with contemporary cardio-oncology guidelines, the recognition that subclinical reductions in ventricular function carry clinical significance further emphasizes the importance of incorporating GLS assessment into routine evaluation in order to enable earlier detection of cardiac injury and guide timely interventions.

Finally, the increased incidence of renal toxicity among allogeneic HSCT recipients underscores the need for integrated renal and cardiovascular monitoring, given the interplay between kidney and heart function in this population. Overall, these findings emphasize the importance of multimodal, risk-adapted cardiac surveillance to improve long-term outcomes in HSCT recipients.

## Figures and Tables

**Table 1:** Baseline characteristics of HSCT recipients.

Variable	Total (*n* = 518)	Allogeneic HSCT (*n* = 454)	Autologous HSCT (*n* = 64)
Age ≥ 60 years	139 (26.8%)	117 (25.8%)	22 (34.4%)
Male sex	302 (58.3%)	264 (58.1%)	38 (59.4%)
Smoking history	228 (44.0%)	213 (46.9%)	15 (23.4%)
Family history of CV disease	58 (11.2%)	51 (11.2%)	7 (10.9%)
Hypertension	143 (27.6%)	124 (27.3%)	19 (29.7%)
Diabetes mellitus	31 (6%)	26 (5.7%)	5 (7.8%)
Hypercholesterolemia	69 (13.3%)	61 (13.4%)	8 (12.5%)
BMI ≥ 25 kg/m^2^	155 (29.9%)	135 (29.7%)	20 (31.2%)
Chronic kidney disease	21 (4.1%)	20 (4.5%)	1 (1.6%)
Pre-HSCT atrial fibrillation	20 (3.9%)	13 (2.9%)	7 (10.9%)
LVEF < 55%	26 (5%)	23 (5.1%)	3 (4.7%)
GLS > −20% *	50/338 (14.7%)	44/288 (15.2%)	6/50 (12%)

* GLS values available in 338 patients. CV: Cardiovascular; BMI: Body Mass Index; HSCT: Hematopoietic Stem Cell Transplantation; LVEF: Left Ventricular Ejection Fraction; GLS: Global Longitudinal Strain.

**Table 2:** Baseline characteristics between patients without GLS and patients with GLS.

Variable	No GLS Available (*n* = 180)	GLS Available (*n* = 338)	*p*
Age, years	49.8 ± 12.3	50.4 ± 12.7	0.593
Age ≥ 60 years	39/180 (21.6%)	91/338 (26.9%)	0.238
Male sex	100/180 (55.5%)	194/338 (57.4%)	0.802
Autologous HSCT	16/180 (8.8%)	48/338 (14.2%)	0.048
Allogeneic HSCT	164/180 (91.1%)	290/338 (85.8%)	0.374
Smoking history	81/180 (45%)	157/338 (46.4%)	0.861
Hypertension	61/180 (33.9%)	116/338 (34.3%)	1.000
Diabetes mellitus	12/180 (6.7%)	28/338 (8.3%)	0.639
Hypercholesterolemia	42/180 (23.7%)	58/338 (17.1%)	0.108
Overweight/obesity coded	42/180 (23.3%)	111/338 (32.8%)	0.033
Chronic kidney disease	10/180 (5.5%)	11/338 (3.2%)	0.950
Pre-HSCT AF	7/180 (3.9%)	13/338 (3.8%)	1.000
Overall CV composite	55/180 (30.5%)	106/338 (31.3%)	0.588

HSCT: Hematopoietic Stem Cell Transplantation; AF: Atrial Fibrillation.

**Table 3:** Cardiovascular events between patients in 2004–2014 and patients in 2015–2025.

Variable	2004–2014 (*n* = 51)	2015–2025 (*n* = 467)	*p*
Overall CV composite	17/51 (33.3%)	144/467 (30.8%)	0.653
MACE	6/51 (11.7%)	26/467 (5.6%)	0.119
CV composite excluding Pericardial Effusion	16/51 (31.3%)	125/467 (26.7%)	0.444
AF/flutter	6/51 (11.7%)	60/467 (12.8%)	1.000
Pericardial effusion	14/51 (27.4%)	67/467 (14.3%)	0.014
Reduced LVEF	7/51 (13.7%)	56/467 (11.9%)	0.788
MI	3/51 (5.8%)	7/467 (1.5%)	0.058
Stroke	0/51 (0.0%)	6/467 (1.3%)	1.000
CV death	3/51 (5.8%)	18/467 (3.9%)	0.435
PE	3/51 (5.8%)	21/467 (4.5%)	0.485
GLS available	19/51 (37.2%)	319/467 (68.3%)	<0.001

CV: Cardiovascular; MACE: Major Adverse Cardiovascular Events; AF: Atrial Fibrillation; LVEF: Left Ventricular Ejection Fraction; MI: Myocardial Infarction; PE: Pulmonary Embolism; GLS: Global Longitudinal Strain.

**Table 4:** Underlying disease and type of transplantation.

Disease	Overall *n* (%)	Auto-HSCT	Allo-HSCT
AML	228 (44.0%)	8	220
ALL	81 (15.6%)	4	77
Lymphoma	58 (11.2%)	20	38
MDS	44 (8.5%)	1	43
MPN	42 (8.1%)	0	42
Multiple myeloma	41 (7.9%)	28	13
CLL	13 (2.5%)	0	13
CML	7 (1.4%)	0	7
Multiple sclerosis	3 (0.6%)	3	0
Ewing sarcoma	1 (0.2%)	0	1

AML: Acute Myeloid Leukemia; ALL: Acute Lymphoblastic Leukemia; MDS: Myelodysplastic Syndromes; MPN: Myeloproliferative Neoplasms; CLL: Chronic Lymphoblastic Leukemia; CML: Chronic Myeloid Leukemia.

**Table 5:** Conditioning regimen among allogeneic recipients.

Variable	RIC (*n* = 188)	MAC (*n* = 266)	*p*
Age ≥ 60 years	94 (50.0%)	23 (8.6%)	<0.001
Male sex	109 (58.0%)	155 (58.3%)	0.95
Smoking history	62 (33.0%)	119 (44.7%)	0.01
Hypertension	61 (32.4%)	61 (22.9%)	0.02
Hypercholesterolemia	32 (17.0%)	29 (10.9%)	0.05
Chronic kidney disease	15 (7.9%)	6 (2.3%)	0.01
Pre-HSCT AF	8 (4.3%)	5 (1.9%)	0.15

RIC: Reduced Intensity Conditioning; MAC: Myeloablative Conditioning; HSCT: Hematopoietic Stem Cell Transplantation; AF: Atrial Fibrillation.

**Table 6:** Cardiovascular outcomes: (**A**) by transplant type; (**B**) by conditioning regimen.

***A***			
Total cardiovascular events = 319	Auto-HSCT (*n* = 64)	Allo-HSCT (*n* = 454)	*p*
CV death	1 (1.6%)	12 (2.6%)	0.64
Myocardial infarction	0	11 (2.4%)	0.30
Stroke	0	6 (1.3%)	0.55
Atrial fibrillation	11 (17.2%)	56 (12.3%)	0.34
Pericardial effusion	7 (10.9%)	72 (15.9%)	0.34
Reduced LVEF	14 (21.9%)	62 (13.6%)	0.07
Pulmonary embolism	1 (1.6%)	3 (0.7%)	0.42
Acute kidney injury	2 (3.1%)	61 (13.4%)	0.03
***B***			
Event	RIC (*n* = 188)	MAC (*n* = 266)	
CV death	7 (3.5%)	6 (2.2%)	
Myocardial infarction	5 (2.5%)	5 (1.8%)	
Stroke	1 (0.5%)	2 (0.7%)	
Atrial fibrillation	28 (14.1%)	28 (10.3%)	
Pericardial effusion	30 (15.1%)	42 (15.5%)	
Reduced LVEF	32 (17.0%)	34 (12.8%)	
Pulmonary embolism	2 (1.0%)	5 (1.8%)	
Acute kidney injury	27 (14.4%)	34 (12.7%)	

HSCT: Hematopoietic Stem Cell Transplantation; CV: Cardiovascular; LVEF: Left Ventricular Ejection Fraction; RIC: Reduced Intensity Conditioning; MAC: Myeloablative Conditioning.

**Table 7:** Predictors of GLS deterioration and GVHD: (**A**) predictors of GLS deterioration. (**B**) predictors of GVHD.

***A***			
**Variable**	**OR**	**95% CI**	***p***
RIC vs. MAC	2.1	1.24–3.54	0.006
Age	1.21	1.05–1.41	0.01
***B***			
**Variable**	**OR**	**95% CI**	***p***
Cardiovascular events	1.68	1.15–2.46	0.008
Acute kidney injury	2.2	1.13–3.78	0.03
Pulmonary embolism	4.32	2.48–7.53	<0.001
Overweight	3.4	1.80–6.45	<0.001

RIC: Reduced Intensity Conditioning; MAC: Myeloablative Conditioning.

**Table 8:** Multivariable logistic regression model for cardiovascular events.

Variable	OR	95% CI	*p*
Age (per 10 years)	1.28	1.10–1.48	0.002
Male sex	1.12	0.82–1.53	0.48
Allogeneic HSCT	1.31	0.81–2.11	0.27
Hypertension	1.34	0.98–1.82	0.07
Diabetes	1.56	0.88–2.76	0.12
CKD	2.44	1.18–5.02	0.01
Pre-HSCT AF	2.12	1.04–4.31	0.03
BMI ≥ 25	1.08	0.80–1.46	0.61
Smoking	1.16	0.87–1.54	0.32
Baseline GLS > −20%	1.89	1.02–3.52	0.04

HSCT: Hematopoietic Stem Cell Transplantation; CKD: Chronic Kidney Disease; AF: Atrial Fibrillation; BMI: Body Mass Index; GLS: Global Longitudinal Strain.

## Data Availability

The data presented in this study are available upon reasonable request from the corresponding author. The data are stored in the electronic registries of the internal network of Azienda Ospedaliero-Universitaria Careggi and are not publicly available due to privacy and ethical restrictions.
